# The conundrum of metaplastic breast cancer: a single Egyptian institution retrospective 10-year experience (2011–2020)

**DOI:** 10.1186/s43046-023-00178-z

**Published:** 2023-06-05

**Authors:** Yahia Ismail, Amr Kamal, Rasha Allam, Al-Shimaa Zakaria

**Affiliations:** 1 Medical Oncology Department, National Cancer Institute, Cairo University, Cairo, 11796 Egypt; 2 Surgical Oncology Department, National Cancer Institute, Cairo University, Cairo, 11796 Egypt; 3 Cancer Epidemiology & Biostatistics Department, National Cancer Institute, Cairo University, Cairo, 11796 Egypt; 4 Pathology Department, National Cancer Institute, Cairo University, Cairo, 11796 Egypt

**Keywords:** Metaplastic, Breast cancer, Triple negative, National Cancer Institute, Egypt

## Abstract

**Background:**

Metaplastic breast cancer (MetBC) still represents a conundrum owing to its peculiar histogenesis and molecular drivers that render it extremely resistant to standard chemotherapy with ultimate dismal survival.

**Aim:**

Describe the Egyptian National Cancer Institute’s (NCI-E) experience with MetBC regarding its clinicopathologic features, treatment, and survival outcomes.

**Patients and methods:**

Between 2011 and 2020, all MetBC patients presented to NCI-E were retrospectively evaluated. Original clinicopathologic data, therapeutic modalities, pathologic response to neoadjuvant chemotherapy (NACT), recurrence, and date of last follow-up/death were obtained from archived charts.

**Results:**

A cohort of 135 females, the median age was 52 years, and median follow-up period was 40 months (range: 2.6–130.8). Two-thirds were triple negative (TN). Squamous carcinoma was prevalent in 74.8% followed by carcinoma with osseous/chondroid differentiation, spindle cell, and low-grade adenosquamous carcinoma encountered in 13.3, 7.4, and 4.5%, respectively. Modified radical mastectomy was done in 59.3%, and positive nodes (pN+) were depicted in 37.7%. Median Ki-67 was 45% (range: 10–88); grade III and lymphovascular invasion (LVI) were observed in 83.7 and 43.7%, respectively. Stage II was the most common (49%), whereas initial stage IV was encountered in 8.1%. Anthracyclines/taxane combinations were rampant in adjuvant/neoadjuvant settings. The latter was employed in 41 patients, with only 3 cases (7.3%) achieving pathologic complete response (pCR), while moderate/significant residual tumor burden was found in 83%. The 5-year DFS and OS were 56.4 and 57.6%, respectively. Spindle cell carcinoma showed the worst survival parameters in univariate analysis. On the multivariate level, higher tumor stage (pT3 & 4), Ki-67 ≥ 45%, and TN subtype were independent variables for worse DFS and OS; age ≥ 52 years and the presence of LVI were independent features for worse DFS, whereas pN+ was an independent parameter for worse OS.

**Conclusions:**

This study further solidifies the dreadful response of MetBC to conventional chemotherapy regimens employed in common non-metaplastic pathologies. A radical shift in treatment standards tailored to combat the molecular landscape of this distinctive tumor is urgently needed. Immunotherapy and molecularly targeted agents demonstrated promising results in phase I and II trials with hopeful sooner implementation in phase III studies.

## Introduction

Metaplastic breast cancer (MetBC) is a rare variant of primary malignant breast tumors that accounts for about 0.2–1% worldwide, and despite its rarity, it conveys a grave prognosis compared to other breast cancer (BC) varieties [1]. It represented 0.7% of primary invasive BC in adults according to the National Cancer Institute of Egypt (NCI-E) Cancer Pathology Registry for 12 years (2000–2011) [2]. Histologically, it arises due to the conversion of a portion or the entire carcinomatous glandular element of the breast tumor to a non-glandular epithelial entity like squamous cell carcinoma or mesenchymal (sarcomatous) constituents [3, 4]; accordingly, the WHO categorizes MetBC as squamous cell carcinoma, spindle cell carcinoma, low-grade adenosquamous carcinoma, fibromatosis-like, mixed metaplastic and metaplastic carcinoma with mesenchymal differentiation (e.g., chondroid, osseous) [1].

In an attempt to elucidate the justification for the synchronicity of the carcinomatous and sarcomatous histologies in an individual tumor, three theoretical models were postulated: firstly, the conversion (metaplastic) theory conceptualizes that the sarcomatous constituents arise through progressive metaplastic alterations of the carcinomatous components, whereas the second (collision) model proposes that both elements are originating from discrete progenitor cells and then unite to form a single tumor. The third (combination) theory advocates the unified source of both components from a multipotent progenitor cell [5]. Immunohistochemistry (IHC) could be a further confirmatory process for the metaplastic theory by the detection of a myoepithelial marker like S-100, a mesenchymal one as vimentin, and/or an epithelial marker (cytokeratin) within an individual tumor expressed in both sarcomatous and carcinomatous portions [6].

The majority of MetBC tumors express the triple-negative (TN) molecular subtype [7], and as most of the metaplastic variants recognized by the WHO are resistant to chemotherapy and extremely aggressive except the low-grade adenosquamous and fibromatosis-like carcinomas [8], metaplastic TN patients almost carry double the hazard of relapse with ultimately shorter survival parameters in comparison with their non-metaplastic counterparts with an estimated median survival of 8 months for patients with distant metastases [9, 10].

Despite the conspicuous striking evidence from numerous studies in the literature demonstrating that conventional neoadjuvant chemotherapy (NACT) has remarkably poor efficacy for decreasing the cancer burden with the eventual disappointing outcome, still the current standards of care for MetBC are following the same strategies applied for TN-invasive duct carcinoma (IDC) patients [11]. Hennessy et al. [12] reported only a 10% pathologic complete response (pCR) rate among metaplastic patients who received preoperative chemotherapy, and in the study of Chen et al. [13], the progression rate was 83% in patients who received NACT. Aydiner et al. [14] identified a response of 12.5% for the MetBC patients who received neoadjuvant anthracycline and taxane versus 75% in the TN non-metaplastic group with a 0% pCR rate in the former group. The latter dreadful 0% of pCR was also described in the reports of Zhang et al. [15] and Corso et al. [16]. The same upsetting response rate was also encountered in the metastatic setting, as Chen et al. [13] reported 8.3% and Cardoso et al. [17] described a response of 16.7% in metastatic MetBC cases compared to 21–75% in metastatic IDC patients.

Exploration of the possible underlying molecular mechanisms that might explain the hostile nature of this tumor, the resistance to conventional chemotherapy, and the propensity for early metastases were explicated in multiple studies, e.g., the role of the epithelial-to-mesenchymal transition (EMT) [18], the phosphoinositide 3-kinase (PI3K) pathway hyperactivity [19, 20], the role of the stem-cell-like features [21], the hyperactivation of the EGFR signalling cascade [22], the nitric oxide synthase (NOS) signalling pathway [23], and the programmed death ligand-1 (PD-L1) overexpression [24]. Subsequently, tremendous efforts are being exerted in multiple phase I and II trials trying to implement new treatment approaches targeting the molecular machinery explored, attempting to radically change the treatment landscape and overcome the resistance of this devastating type of BC with promising results; nevertheless, none of these drugs is assigned as category 1 recommendation so far [11]. Herein, we present our institute's a 10-year experience and outcome of this peculiar aggressive form of BC.

## Patients and methods

Between January 2011 and December 2020, all patients with the diagnosis of MetBC presented to NCI-E were retrospectively evaluated. The eligibility criteria were as follows: adult females aged ≥ 18 years with confirmed pathological diagnosis of MetBC, any stage of the disease at presentation, and complete follow-up data in the patient’s chart. After searching the database of the pathology department, 143 cases were found to have the desired diagnosis during the specified 10-year period; on retrieving their archived medical files, 8 patients were excluded due to early loss of follow-up or insufficient data; hence, the retrospective descriptive and survival analyses were carried out for the remaining 135 cases. The demographic and clinicopathological data included: age at diagnosis; menopausal status; family history; the histological subtype of MetBC according to the WHO classification of breast tumors [1]; tumor grade; TNM stage as per the 8th edition of AJCC [25]; breast cancer molecular subtype determined upon ER, PR, HER2, and Ki-67 [26]; date and type of surgery; therapy employed (chemotherapy either adjuvant/neoadjuvant or palliative,anti-HER2, hormonal treatment, radiation therapy, etc.); the pathologic response for patients who received NACT assessed by the residual cancer burden tool (RCB) [27]; date and site of recurrence; and date of last follow-up/death were extracted from the archived patients’ files. The data collection cut-off date was on June 30, 2022.

### Statistical methodology

Data management and analyses were done using Statistical Package for Social Sciences (SPSS) v.24. Data were expressed as mean ± standard deviation (SD), median (range), or number (%) as appropriate. *p* < 0.05 indicates statistical significance. All tests were two-sided. Chi-square or Fisher’s tests were used to compare the independent groups concerning the categorical data, as appropriate. Kaplan-Meier method was employed to estimate the disease-free survival (DFS) and overall survival (OS). Prognostic variables were related to survival using the log-rank test. Parameters with a significance level < 0.10 on univariate level were selected to enter the stepwise Cox regression model. The latter was used to estimate the hazard ratio (HR) and 95% confidence interval (95% CI). DFS was calculated as the time from the date of curative surgery until the date of recurrence (local or distant), death, or last follow-up, and OS was measured from the date of diagnosis to the date of death or last follow-up.

## Results

### Clinicopathologic features

**Fig. 1:**
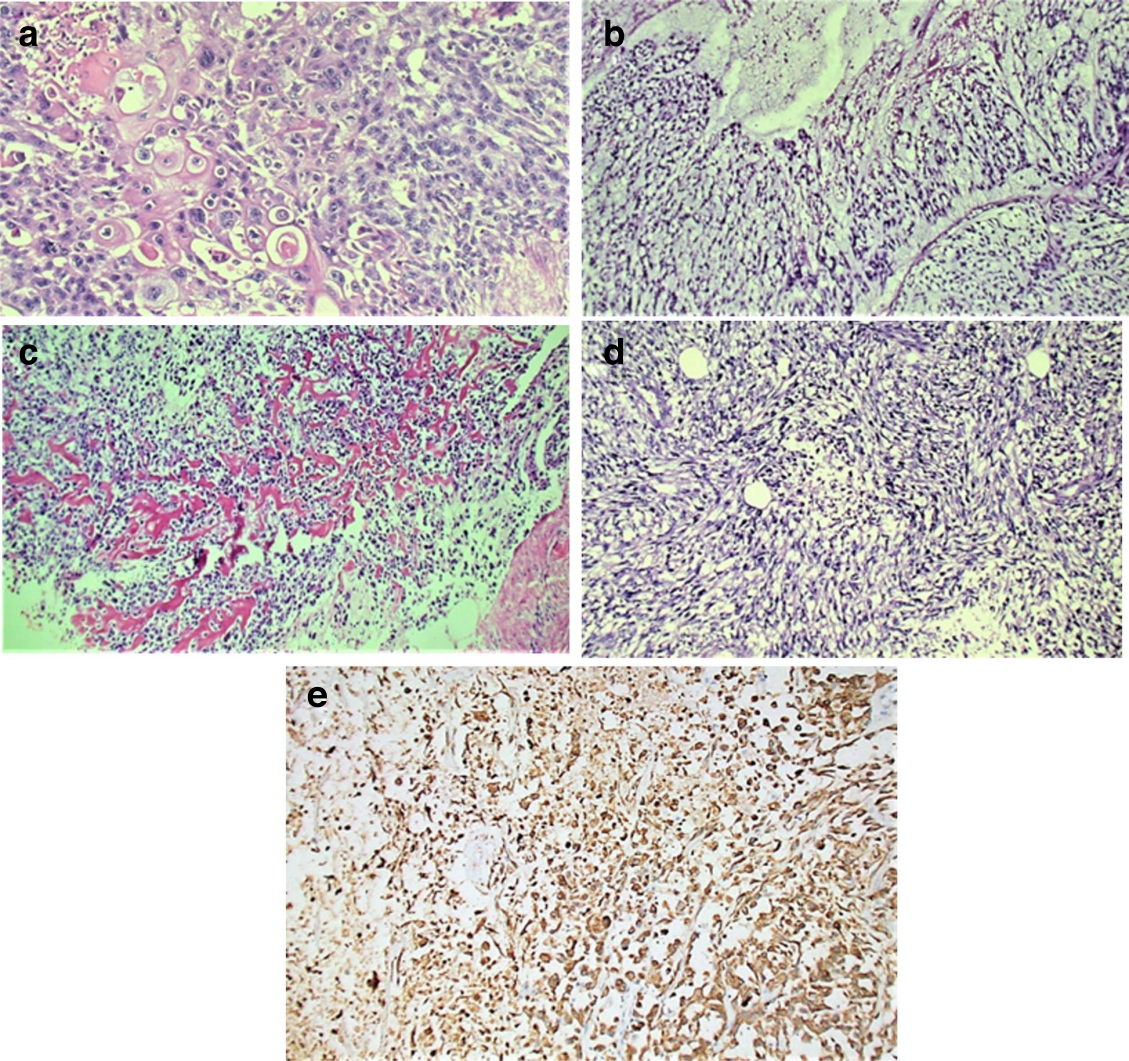
**a** Metaplastic breast squamous cell carcinoma shows nests of malignant squamous cells with keratin pearls formation (hematoxylin and eosin, original magnification × 400). **b** Metaplastic breast carcinoma shows groups of malignant cells with chondroid and myxoid background (chondroid differentiation) (hematoxylin and eosin, original magnification × 200). **c** Metaplastic breast carcinoma shows sheets of malignant cells with areas of osteoid-like material (osseous differentiation) (hematoxylin and eosin, original magnification × 200). **d** Metaplastic breast spindle cell carcinoma shows spindle cell morphology of the tumor cells (hematoxylin and eosin, original magnification ×400). **e** Metaplastic breast carcinoma shows positive reaction to cytokeratin in the epitheloid and the spindle cells (cytokeratin immunostaining, original magnification ×400)

A total of 135 female patients with a median age of 52 years (range: 22–88), the majority (59.3%) were postmenopausal at initial diagnosis, pT2 was prevalent in 45.9% of patients, and pathologically positive axillary lymph nodes (pN+) were depicted in only 51 patients (37.7%) with capsular invasion encountered in two-thirds of them. Almost half of the cases (49%) presented with TNM stage II, whereas initially metastatic disease (stage IV) was encountered in just 11 patients (8.1%), with the lung being the most common site of original spread found in 91% of those patients. The median Ki-67 proliferation index was 45% (range: 10–88); grade III and lymphovascular invasion were found in 83.7 and 43.7%, respectively. The most popular molecular subtype was the TN depicted in two-thirds (66%) of the group, whereas HER2 was overexpressed in a minor fraction (16.3%) (Table 1). Squamous cell carcinoma was the major histological subtype presented in almost three-quarters of the patients (74.8%), followed by carcinoma with osseous and/or chondroid differentiation, spindle cell carcinoma, and low-grade adenosquamous carcinoma encountered in 13.3, 7.4, and 4.5%, respectively (Fig. 1).

**Table 1:** Clinicopathologic features of 135 metaplastic breast cancer patients

**Characteristic**	***n* (%)**
Age at diagnosis (y)	
Median (range); mean ± SD	52 (22–88); 52.27 ± 13.91
Menopausal status	
Premenopausal	55 (40.7)
Postmenopausal	80 (59.3)
Laterality	
Right breast	64 (47.4)
Left breast	71 (52.6)
pT size^a^ (cm); median (range)	4(1–17)
≤ 4 cm	65 (48.1)
> 4 cm	60 (44.5)
Unknown	10 (7.4)
pT stage	
T1	8 (5.9)
T2	62 (45.9)
T3	24 (17.8)
T4	31 (23.0)
Unknown	10 (7.4)
pN stage	
N0	68 (50.4)
N1	29 (21.5)
N2	18 (13.3)
N3	4 (3.0)
Unknown	16 (11.8)
pN+; median(range) (*n* = 51)	3 (1–18)
Capsular invasion in pN+ patients (*n* = 51)	
Present	34 (66.7)
Absent	17 (33.3)
LVI	
Present	59 (43.7)
Absent	74 (54.8)
Unknown	2 (1.5)
Tumor grade	
II	22 (16.3)
III	113 (83.7)
Initial TNM stage	
Stage I	5 (3.7)
Stage II	66 (49.0)
Stage III	47 (34.8)
Stage IV	11 (8.1)
Unknown	6 (4.4)
Ki-67%, median(range)	45 (10–88)
≤ 45%	76 (56.3)
> 45%	53 (39.3)
Unknown	6 (4.4)
ER status	
Negative	112 (83.0)
Positive	23 (17.0)
PR status	
Negative	107 (79.3)
Positive	28 (20.7)
HER-2 status	
Negative	113 (83.7)
Positive	22 (16.3)
Molecular subtype	
LA	1 (0.7)
LB	31 (23.0)
HER2 enriched	14 (10.3)
TN	89 (66.0)
**Histologic subtype of MetBC**	
Squamous cell carcinoma	101 (74.8)
Carcinoma with chondroid and/or osseous differentiation	18 (13.3)
Spindle cell carcinoma	10 (7.4)
Low-grade adenosquamous carcinoma	6 (4.5)
**Recurrence after curative surgery (*n* = 119)**	
Yes	41 (34.5)
No	78 (65.5)
**Recurrence sites (*n* = 41)**	
Lung ± visceral/bone/brain metastases	24 (58.5)
Locoregional recurrence only	11 (26.8)
Contralateral breast	4 (9.8)
Bone only	2 (4.9)

^a^Largest tumor diameter, *cm* centimeter, *ER* estrogen receptor, *HER2* human epidermal growth factor-2, *LA* luminal A, *LB* luminal B, *LVI* lymphovascular invasion, *MetBC* metaplastic breast cancer, *n* number, *pT* pathologic tumor stage, *pN* pathologic nodal stage, *SD* standard deviation, *TN* triple negative, *TNM* tumor-node metastasis, *y* year

### Treatment modalities employed

Curative surgery was employed in the majority of the cohort (119 patients; 88.1%), with modified radical mastectomy (MRM) as the commonest in 59.2%, followed by breast conservation surgery (BCS) in 28.9% of the patients. Palliative mastectomy was carried out in 6 (4.4%) patients due to tumor fungation/bleeding. The vast majority of patients who underwent curative surgery were candidates for adjuvant chemotherapy (80 cases; 59.25%), being locally advanced at presentation before receiving NACT, or patients with pathologically positive nodes and/or with pathological tumor size > 5 cm. The combinations of anthracyclines and taxanes regimens were the rampant protocols given to 40 (50%) patients. Anthracyclines only were employed in 20 cases (25%), taxanes only in 14 patients (17.5%), and adjuvant capecitabine was given to only five TN patients (6.3%) with residual disease after NACT. Adjuvant trastuzumab was delivered to 14 patients (10.4%) with HER2 amplification, whereas adjuvant hormonal therapy was delivered to 30 (22.2%) luminal cases, and postoperative radiation therapy was given to 79% of the cohort. Concerning the neoadjuvant treatment, forty-one patients (30.4%) received NACT, with approximately two-thirds of them got anthracyclines and taxanes combination protocols; meanwhile, platinum-containing regimens were given to only 4 (9.7%) cases. Pathologic complete response (pCR) after NACT was achieved in only 3 (7.3%) cases (RCB-0), whereas moderate and significant residual tumor burden (RCB-II & III) were encountered in 83% (Table 2). All the three patients who accomplished pCR had squamous cell carcinoma histology, two of them had TN subtype, and the third had LB with HER2 overexpression (Table 3).

**Table 2:** Treatment employed in 135 metaplastic breast cancer patients

**Treatment modality**	***n* (%)**
Surgery type	
MRM	80 (59.3)
BCS	39 (28.9)
Palliative mastectomy	6 (4.4)
No surgery	10 (7.4)
NACT	
Yes	41 (30.4)
No	94 (69.8)
^a^NACT type (*n* = 41)	
Comb. anthracycline & taxanes	27 (65.0)
Anthracycline only	11(26.8)
Platinum-containing regimen	4 (9.7)
Others	1 (2.4)
Pathologic Response to NACT(*n* = 41)	
RCB-0	3 (7.3)
RCB-I	3 (7.3)
RCB-II	14 (34.1)
RCB-III	20 (48.9)
Unknown	1 (2.4)
ACT	
Yes	80 (59.3)
No	55 (40.7)
ACT type (*n* = 80)	
Comb. anthracycline and taxanes	40 (50.0)
Anthracycline only	20 (25.0)
Taxanes only	14 (17.5)
Capecitabine	5 (6.25)
Others	1 (1.25)
Adjuvant trastuzumab for HER2+ cases (*n* = 19)	
Yes	14 (73.7)
No	5 (26.3)
Adjuvant hormonal for luminal cases (*n* = 32)	
Yes	30 (93.7)
No	2 (6.3)
Adjuvant hormonal type (*n* = 30)	
Tam	19 (63.3)
AI	9 (30)
Tam followed by AI	2 (6.7)
Adjuvant radiation therapy (*n* = 119)	
Yes	94 (79)
No	25 (21)

^a^Two patients received combination of anthracyclines, taxanes, and platinum, *ACT* adjuvant chemotherapy, *AI* aromatase inhibitor, *BCS* breast-conserving surgery, *Comb*. combination, *MRM* modified radical mastectomy, *n* number, *NACT* neoadjuvant chemotherapy, *RCB* residual cancer burden, *Tam* tamoxifen

**Table 3:** Characteristics of 3 cases achieved pCR after neoadjuvant chemotherapy

Case no.	Age (y)	Metaplastic subtype	Grade	Molecular subtype	NACT regimen	PORT	DFS (ms)	OS (ms)
1	55	SCC	III	LB-HER2+	TCH × 6	Yes	12.63	25.72
2	56	SCC	III	TN	AC × 4-Tw × 12	Yes	17.8	25.3
3	35	SCC	III	TN	FAC × 4-Tax/Carb × 4	Yes	46.88	56.09

*AC* adriamycin and cyclophosphamide, *FAC* fluorouracil, adriamycin, and cyclophosphamide, *LB-HER2+* luminal B with HER2 overexpression, *ms* months, *NACT* neoadjuvant chemotherapy, *PORT* postoperative radiation therapy, *SCC* squamous cell carcinoma, *Tax/Carb* taxol and carboplatin, *TCH* docetaxel, carboplatin, and trastuzumab, *TN* triple negative, *Tw* taxol weekly, *y* year

### Comparison between the TN and non-TN cases

Comparing the group of TN patients which represented the majority of the cohort (66%) to those with other molecular subtypes (non-TN; 34%), no statistically significant *p*-values were observed regarding any of the different clinicopathologic categories, treatment modalities, response to NACT, or the incidence of recurrence.

### Survival analyses

**Fig. 2:**
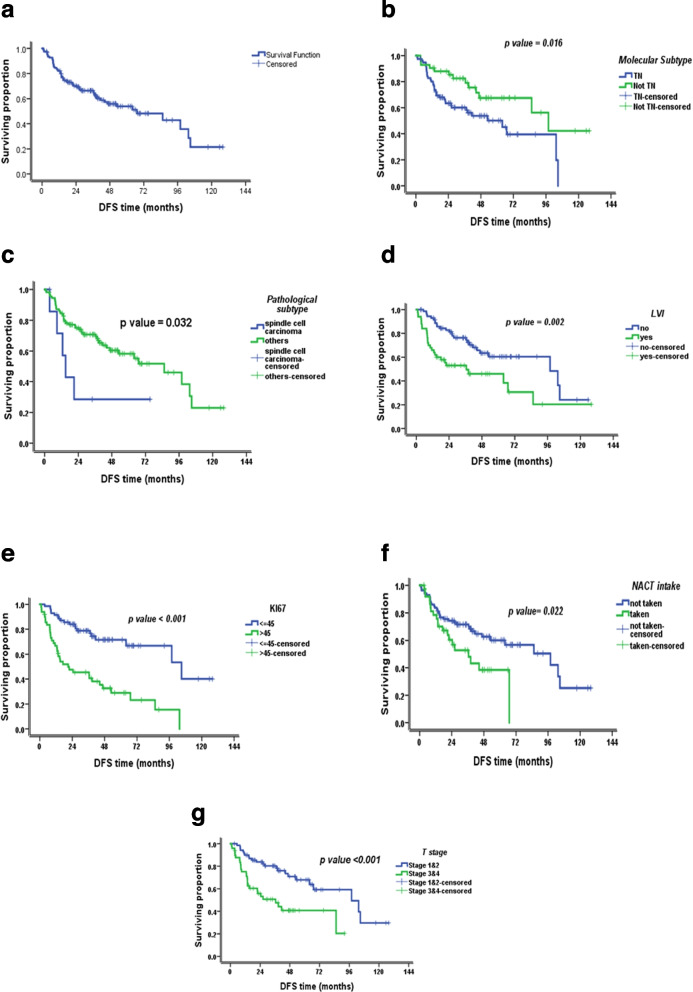
**a** DFS for the whole metaplastic breast cancer cohort. **b** DFS according to the molecular subtype. **c** DFS according to the metaplastic pathological subtype. **d** DFS according to LVI. **e** DFS according to Ki-67(%). **f** DFS according to NACT. **g** DFS according to pT stage

**Fig. 3:**
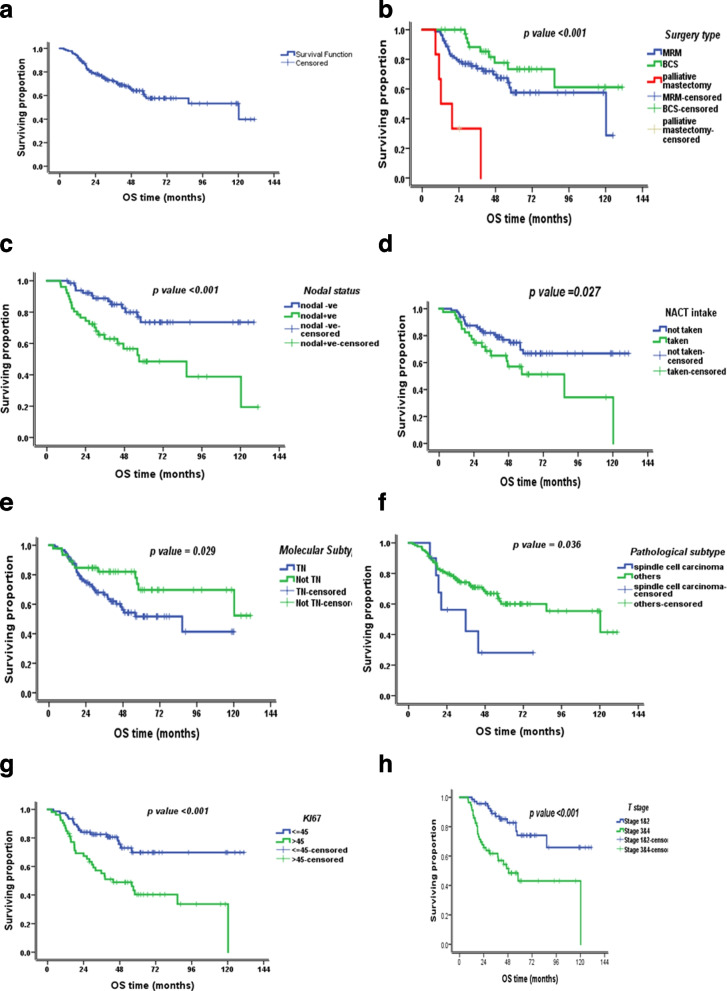
**a** OS for the whole metaplastic breast cancer cohort. **b** OS according to the surgery type. **c** OS according to pathological axillary nodal status. **d** OS according to NACT. **e** OS according to the molecular subtype. **f** OS according to the metaplastic pathological subtype. **g** OS according to Ki-67(%). **h** OS according to pT stage

At the end of the follow-up period (median 40 months; range: 2.6–130.8), disease recurrence after curative surgery was found in 41 out of 119 patients (34.5%); the most common site for relapse was the lung either alone or with other visceral metastases encountered in more than half of the recurrent patients (58.5%), followed by loco-regional recurrence developed in 26.8%; and recurrence in the contralateral breast and bone only was found in 9.8 and 4.9%, respectively (Table 1). The median DFS and OS were 85.4 and 120.3 months, respectively. The cumulative 5-year DFS and OS were 56.4 and 57.6%, respectively, and the cumulative10-year DFS and OS were 22.4 and 53.1%, respectively. On univariate analysis, age ≥ 52 years, maximum tumor diameter ≥ 4 cm, pT stages 3 and 4, pN+, the presence of LVI, Ki-67 ≥ 45%, TN molecular subtype, spindle cell carcinoma histology, receipt of NACT, not receiving ACT, and adjuvant chemotherapy protocols other than anthracycline only were significantly correlated with worse DFS and OS (Figs. 2 and 3). TNM stage III was related to worse DFS, whereas stage IV had the significantly worse OS; there was no statistically significant difference with respect to DFS between patients who underwent MRM vs. BCS. However, the latter showed significantly superior OS compared to MRM or palliative mastectomy (Tables 4 and 5). As regards the multivariate analysis (MVA), advanced pT stage (3 & 4), high Ki-67 ≥ 45%, and TN molecular subtype were independent prognostic variables for worse both DFS and OS. Age ≥ 52 years and the presence of LVI were independent prognostic factors for worse DFS, whereas pN+ was an independent prognostic feature for worse OS (Table 6).

**Table 4:** Univariate analysis for disease-free survival

	Total no.	No. of events	Cumulative survival estimate at 5 years (%)	*p*-value
**Whole group**	**119**	50	0.564	-
**Age (years)**				
< 52	61	22	0.679	**0.036**
≥ 52	58	28	0.435	
**Curative surgery type**				
MRM	80	33	0.562	0.577
BCS	39	17	0.580	
**Maximum tumor diameter (cm)**				
< 4	65	24	0.630	**0.037**
≥ 4	54	26	0.502	
**pT stage**				
Stages 1 & 2	70	23	0.678	**< 0.001**
Stages 3 & 4	49	27	0.407	
**pN status**				
Negative	68	24	0.634	**0.023**
Positive	51	26	0.460	
**Capsular invasion** (*n* = 51)				
Absent	17	6	0.589	0.220
Present	34	20	0.410	
**LVI (*n* = 117)**				
Absent	73	26	0.605	**0.012**
Present	44	23	0.520	
**Grade**				
II	20	8	0.610	0.627
III	99	42	0.558	
**Initial TNM stage**				
Stages I & II	71	24	0.659	**0.001**
Stage III	47	26	0.414	
**Pathological subtype**				
Spindle cell carcinoma	8	5	0.286	**0.032**
Others	111	45	0.582	
**Pathological subtype**				
Squamous cell carcinoma	89	36	0.561	0.853
Others	30	14	0.584	
**Ki-67 %**				
< 45	58	16	0.736	**< 0.001**
≥ 45	55	34	0.359	
**Molecular subtype**				
TN	77	37	0.501	**0.016**
Non-TN	42	13	0.675	
**NACT receipt**				
Not received	78	31	0.632	**0.015**
Received	41	19	0.395	
**NACT type (*n* = 41)**				
Anthracycline/taxanes	27	10	0.474	0.119
Others	14	9	0.256	
**Response to NACT (*n* = 40)**				
RCB 0 & I	6	2	NA	0.079
RCBII & III	34	3	0.725	
**ACT receipt**				
Not received	39	25	0.307	**< 0.001**
Received	80	25	0.693	
**ACT type** (*n* = 80)				
Anthracycline only	20	7	0.820	**0.007**
Anthracycline & taxanes	40	10	0.734	
Others	20	9	NA	
**Start of ACT (days)**				
≤ 30	41	10	0.766	0.186
> 30	39	15	0.603	
**PORT**				
No	25	13	0.563	0.642
Yes	94	37	0.564	

*LVI* lymphovascular invasion, *N* number, *pT* pathologic tumor stage, *pN* pathologic nodal stage, *TN* triple negative, *TNM* tumor-node metastasis, *ACT* adjuvant chemotherapy, *BCS* breast-conserving surgery, *MRM* modified radical mastectomy, *NACT* neoadjuvant chemotherapy, *RCB* residual cancer burden, *PORT* postoperative radiotherapy

**Table 5:** Univariate analysis for overall survival

	Total no.	No. of events	Cumulative survival estimate at 5 years (%)	*p*-value
**Whole cohort**	**135**	49	0.576	-
**Age (years)**				
< 52	66	18	0.669	**0.011**
≥ 52	69	31	0.478	
**Surgery type** (*n* = 125)				
MRM	80	27	0.575	**< 0.001**
BCS	39	9	0.734	
Palliative mastectomy	6	5	NA	
**Maximum tumor diameter** (cm) (*n* = 125)				
< 4	65	14	0.740	**< 0.001**
≥ 4	60	27	0.445	
**pT stage** (*n* = 125)				
Stages 1 & 2	70	14	0.741	**< 0.001**
Stages 3 & 4	55	27	0.436	
**pN status** (*n* = 119)				
Negative	68	13	0.735	**0.001**
Positive	51	24	0.485	
**Capsular invasion** (*n* = 51)				
Absent	17	6	0.676	0.186
Present	34	18	0.403	
**LVI** (*n* = 133)				
Absent	74	17	0.700	**< 0.001**
Present	59	31	0.413	
**Grade**				
II	22	6	0.693	0.279
III	113	43	0.549	
**Initial TNM stage** (*n* = 129)				
Stages I &II	71	0	0.718	**< 0.001**
Stage III	47	21	0.508	
Stage IV	11	8	0.182	
**Pathological subtype**				
Spindle cell carcinoma	10	6	0.281	**0.036**
Others	125	43	0.600	
**Pathological subtype**				
Squamous cell carcinoma	101	38	0.541	
Others	34	11	0.666	0.263
**Ki-67%** (*n* = 129)				
< 45	64	14	0.730	**< 0.001**
≥ 45	65	35	0.423	
**Molecular subtype**				
TN	89	37	0.517	**0.029**
Non-TN	46	12	0.697	
**NACT receipt**				
Not received	81	21	0.601	**0.027**
Received	41	18	0.513	
**NACT type** (*N* = 41)				
Anthracycline & taxanes	27	9	0.577	0.268
Others	14	9	0.408	
**Response to NACT** (*n* = 40)				
RCB-0 & I	6	1	0.750	0.286
RCB-II & III	34	16	0.487	
**ACT receipt**				
Not received	40	17	0.480	**0.019**
Received	80	20	0.699	
**ACT type** (*n* = 80)				
Anthracycline	20	2	0.868	**0.011**
Anthracycline & taxanes	40	9	0.686	
Others	20	9	0.533	
**ACT start** (days) (*n* = 80)				
≤ 30 days	41	9	0.739	0.716
> 30 days	39	10	0.670	
**PORT** (*n* = 119)				
No	25	10	0.578	0.253
Yes	94	26	0.648	

*ACT* adjuvant chemotherapy, *BCS* breast-conserving surgery, *LVI* lymphovascular invasion, *N* number, *pT* pathologic tumor stage, *pN* pathologic nodal stage, *TN* triple negative, *TNM* tumor-node metastasis, *MRM* modified radical mastectomy, *NACT* neoadjuvant chemotherapy, *RCB* residual cancer burden, *PORT* postoperative radiotherapy

**Table 6:** Multivariate analysis using Cox regression hazard model for DFS and OS

	**Beta coefficient**	**Standard error**	***p*-value**	**Hazard ratio**	**95.0% *CI* for hazard ratio**	
					**Lower boundary**	**Upper boundary**
**Disease-free survival**						
Age (years) (≥ 52 vs < 52)	0.733	0.309	**0.018**	2.081	1.137	3.810
pT stage (3 & 4 vs 1 & 2)	0.677	0.317	**0.032**	1.968	1.058	3.660
Ki-67% (≥ 45 vs < 45)	1.127	0.321	**< 0.001**	3.085	1.645	5.788
Molecular subtype (TN vs non-TN)	1.165	0.350	**0.001**	3.205	1.615	6.362
LVI (present vs absent)	0.814	0.311	**0.009**	2.257	1.228	4.150
**Overall survival**						
pT stage (3 & 4 vs 1 & 2)	0.735	0.300	**0.014**	2.086	1.159	3.755
Ki-67% (≥ 45 vs < 45)	1.038	0.313	**0.001**	2.825	1.529	5.219
Molecular subtype (TN vs non-TN)	0.963	0.332	**0.004**	2.619	1.366	5.022
Nodal status (positive vs negative)	0.637	0.300	**0.033**	1.891	1.051	3.403

*LVI* lymphovascular invasion, *pT* pathological tumor stage, *TN* triple negative

## Discussion

The numeral of the metaplastic patients in the current series is 135 throughout a 10-year period which is higher than the numbers conveyed by other authors in the literature during a longer period, e.g., Jung et al. [9] described 35 cases during eight years; Chen et al. [13] and Aydiner et al. [14] reported 46 and 54 patients, respectively, through an era of 22 years; Cimino-Mathews et al. [28] described 45 cases over 14 years; and El Zein et al. [29] reported an analysis of 46 patients throughout 22-year period. The sample size in the current series is nearly similar to Tadros et al. [30] who described 132 cases but through a longer period of 23 years and faintly lower than Corso et al. [16] who reported 153 patients in an era of 22 years.

The median age in the present study was 52 years, near to what was previously reported [13, 16, 29, 31] and almost a decade younger than the authors stated [32, 33]. This inconsistency is almost due to differences in sample size and patient characteristics. Most patients were postmenopausal at diagnosis, which concurs with preceding results [16, 30, 32–34]. MRM was the most commonly employed curative surgery that corresponds with other reports [13–15, 29, 31, 33], a finding that could be explained by that as MetBC is one of the most aggressive pathologies with well-known resistance to neoadjuvant standard chemotherapy regimens with consequent low clinical downstaging rates; most surgeons opt to perform a radical surgery rather than to go for breast conservation. Despite less than a third of our cohort (28.9%) underwent BCS, the latter showed significantly superior OS on univariate analysis (*p* < 0.001), and as 92.3% of those patients received postoperative radiation therapy, we do assume similarity to the results of Zhang et al. [35] and Xia L-Y et al. [36] who described that MetBC patients subjected to BCS, and radiation therapy showed significantly better OS than those who underwent a mastectomy.

MetBC has low predilection for axillary nodal spread; this feature was depicted in our series as the major section (57.1%) of patients who underwent curative surgery had pN0. Pathological T2 and TNM stage 2 were prevalent in around half of the cases, 52 and 49%, respectively, typically matching other previous reports [9, 14, 15, 29, 32–34]. Initially, metastatic disease was diagnosed in 8.1% of our cohort, almost similar to Jung et al. [9] who reported 8.6%, and more or less double of what was stated by Cimino-Mathews et al. [28] and Takla et al. [33] who confirmed 4 and 3%, respectively. The lung was the commonest locality of spread either initially or on the subsequent development of recurrence, in agreement with the findings in [33].

Classically consistent with earlier reports demonstrating that MetBC is mostly TNBC [9, 13, 15, 16, 28, 30–33], two-thirds of our cohort (66%) had TN molecular subtype, when we compared the latter to the non-TN cases (34%); we could not elicit any statistically significant difference regarding the multiple clinicopathologic entities, treatment given, pathological response to NACT, or the event of recurrence between the two groups. Nevertheless, harboring a TN subtype was an independent worse prognostic element for both DFS and OS on MVA. Other studies explored the differences in survival between MetBC and non-metaplastic TNBC; the former had significantly inferior OS than the latter [9, 14, 29, 31, 34, 37]. These findings could be attributed to that the metaplastic pathology per se is a landmark for violence and aggression even when compared to the worst molecular subtype of the conventional IDC (TN).

Two other pathologic features could contribute to the harshness of this tumor: the high both histologic grade and proliferation index; GIII tumors were dominant in 83.7% in the current work, which coincides with the findings in [9, 14, 16, 28–30, 32] and an elevated median Ki-67 index in our patients of 45% (range: 10–88), in the work of Aydiner et al. [14]; a remarkably higher median of 70% was reported; moreover, in the results of Corso et al. [16] and Song et al. [31], 93.5 and 90% of their cohorts had levels ≥ 20 and ≥ 14%, respectively.

Squamous cell carcinoma was the main metaplastic histological subtype encountered in almost three-quarters of the current series (74.8%), analogous to previous results reported [9, 16, 32, 33]. Meanwhile, the mixed histology variant was dominant in Chen et al. [13] and Cimino-Mathews et al. [28]. Furthermore, the spindle cell variety was prevalent in [15, 38]. The latter histology was depicted in only 7.4% of our cases; nonetheless, it demonstrated significantly worst DFS and OS compared to other metaplastic varieties on univariate analysis, nearly similar to the conclusions stated by Song et al. [31] and Rakha et al. [38], the latter author documented that the matrix-producing subtype was related to the best survival. On the other side, the results of Tadros et al. [30] showed that squamous carcinoma and heterologous mesenchymal entities were linked with the poorest and best 5-year OS, respectively. The mixed metaplastic kind was correlated with worse DFS and OS in MVA in the work of Takla et al. [33]; however, the metaplastic subtype showed no significant impact on survival in the work of Corso et al. [16]. Accordingly, no definite solid conclusion regarding the best and worst metaplastic subtype so far could be postulated. However, factors other than the latter mostly motivate each tumor’s behavior, e.g., molecular and genetic aberrations, patient, and tumor characteristics.

Forty-one (30.3%) of our patients received NACT. Other authors also confirmed that minor fractions of their cohorts who received preoperative chemotherapy compared to adjuvant chemotherapy [13–16, 28–30, 32, 33], and combination regimens of anthracycline and taxanes were the most common type employed in almost two-thirds of those patients similar to [28, 39]; the majority (83%) of them had moderate and significant residual tumor burden (RCB-II & III) after NACT with only 3 cases (7.3%) who achieved pCR (RCB-0). Our findings of inadequate response to NACT are entirely matching other former studies and further support the concept of resistance of this tumor to conventional chemotherapy, as the pCR rate was 0% [14–16], 6% [39], 9.8% [37], 10% [12, 30], 17% [28], and 39% [32]. Interestingly, our univariate analysis showed that patients who received NACT had worse DFS and OS than those who did not (*p* = 0.01 & 0.02, respectively); nearly identical to what was described by Aydiner et al. [14], we could refer this result to that majority of patients subjected to NACT had a more advanced local disease and the response to chemotherapy was absolutely poor in the main bulk of those patients (83%). The study of Haque et al. [37] emphasized the significance of achieving pCR in this unique tumor, as MetBC patients who attained pCR had meaningfully greater 5-year OS than those with residual disease after NACT (*p* < 0.001), and fascinatingly, there was an identical survival outcome of the former cohort when compared to IDC patients with pCR (*p* = 0.99), the latter finding persisted even after splitting IDC patients to diverse molecular subtypes (TN, HER2-enriched and luminal with *p*-values: 0.91, 0.57 and 0.99, respectively). Adjuvant chemotherapy was employed in 59.3% of our series; the combinations of anthracyclines and taxanes were the most popular, similar to [14, 15, 28, 31]. Adjuvant chemotherapy was associated with significantly longer DFS and OS in univariate analysis (*p* < 0.001 & 0.019, respectively), approximately compatible with other results [28, 38, 40].

The 5-year DFS in the present study was 56.4% higher than what others described [9, 29, 31] and slightly lower than [15, 28, 33], whereas the 5-year OS in the current series was 57.6% near what was reported by [31, 33, 34] and lower than in [9, 14, 15, 28, 29, 32, 39], these discrepancies in survival figures are mostly due to differences in sample size, patients’ characteristics (most of the studies exclude initially metastatic patients) and follow-up durations. Our MVA results were comparable to other previous reports as high pT stage ( ≥ 3) was an independent prognostic factor for worse both DFS and OS, exactly concurring with previous results [16, 30, 31, 33], high Ki-67 was also an independent characteristic for poorer DFS and OS typically matching the results of [31], pN+ was an independent prognostic parameter for inferior OS in agreement with the reports of [30, 31, 39], and LVI was an independent prognostic feature for worse DFS as described by [30, 38], where it was correlated with poor OS in [39]. Age ≥ 52 years in our study was independent factor for worse DFS; in the work of Corso et al. [16], the postmenopausal category was correlated with worse OS.

Therefore, a radical change in the treatment landscape to achieve higher rates of pCR is mandatory by implementing the new molecular targets; in this respect, Basho et al. testified 8 and 12% complete and partial response rates, respectively, in their phase I trial evaluating the combination of the inhibitors of both mTOR and VEGF pathways (everolimus and bevacizumab, respectively), in addition to liposomal doxorubicin in 52 advanced MetBC patients [41]. Also, the safety and efficacy of the combination of immunotherapy by the anti-PD1 (pembrolizumab) and chemotherapy (nab-paclitaxel) in metastatic HER2-negative patients — that would include metaplastic cases — are currently evaluated in a phase II trial [42]; in this regard, Adams S. [43] described a marvellous response to this chemo-immunotherapy practice in a patient with TN spindle cell MetBC with aggressive local recurrence and lung deposits with high PD-L1 expression. The results of the prospective multicenter phase II trial that evaluated nivolumab (anti-PD1) and ipilimumab (anti-CTLA4) combination therapy in a small cohort of 17 patients with advanced MetBC refractory to conventional lines of chemotherapy were recently released; the ORR was 18% with preserved response for > 2 years; the median PFS and OS were 2 and 12 months, respectively; and the authors discovered that metaplastic tumors with low expression of PD-L1, low mutational burden, and lacking tumor-infiltrating lymphocytes had the best response [44].

## Conclusions

Our findings are more or less consistent with previous reports in the literature, further solidifying the evidence that MetBC is extremely resistant to typical chemotherapy protocols with consequent poor therapy response and eventual bleak prognosis. In our cohort, squamous cell carcinoma was the dominant metaplastic subtype; nevertheless, spindle cell carcinoma variant showed the worst survival parameters in univariate analysis. Although MRM was the frequently employed surgery, patients who underwent breast conservation surgery showed superior overall survival. We do anticipate a rapid radical shift in the treatment standards based on the new immunotherapy drugs (e.g., pembrolizumab, nivolumab, and ipilimumab) and other molecularly targeted therapy that showed promising results in phase I and II trials. We also do acknowledge the drawbacks of retrospective studies, but as MetBC is one of the rarest pathologies, retrospectivity is the salvage way to study its behavior and prognostic parameters.

## Data Availability

The datasets used and/or analyzed during the current study are available from the corresponding author on reasonable request.

## Abbreviations

MetBC: Metaplastic breast cancer
pCR: Pathologic complete response
PD-1: Programmed death-1
mTOR: Mammalian target of rapamycin
CTLA-4: Cytotoxic T-lymphocyte-associated antigen-4
VEGF: Vascular endothelial growth factor

## References

[1] Tan PH, Ellis I, Allison K, Brogi E, Fox SB, Lakhani S, et al.; WHO Classification of Tumours Editorial Board. The 2019 World Health Organization classification of tumours of the breast. Histopathology. 2020;77(2):181-185. 10.1111/his.14091. Epub 2020 Jul 29. PMID: 32056259. 10.1111/his.14091 32056259

[2] Mohamed G. Breast tumors. In: Mokhtar N, Asmaa S, Badawy O, Khorshed E, Mohamed G, Ibrahim M, Abdelazim H, editors. Cancer pathology registry 2000–2011. Cairo: Cairo Press; 2016. Chapter II, p.7–31.

[3] Kaufman MW, Marti JR, Gallager HS, Hoehn JL. Carcinoma of the breast with pseudosarcomatous metaplasia. Cancer. 1984;53:1908–17. 6322962 10.1002/1097-0142(19840501)53:9<1908::aid-cncr2820530917>3.0.co;2-f

[4] Oberman HA. Metaplastic carcinoma of the breast: a clinicopathologic study of 29 patients. Am J Surg Pathol. 1987;11(12):918–29. 2825549 10.1097/00000478-198712000-00002

[5] Zhuang Z, Lininger RA, Man YG, Albuquerque A, Merino MJ, Tavassoli FA. Identical clonality of both components of mammary carcinosarcoma with differential loss of heterozygosity. Mod Pathol. 1997;10(4):354–62 (PMID: 9110298). 9110298

[6] Chhieng C, Cranor M, Lesser ME, Rosen PP. Metaplastic carcinoma of the breast with osteocartilaginous heterologous elements. Am J Surg Pathol. 1998;22:188–94. 9500219 10.1097/00000478-199802000-00006

[7] Weigelt B, Kreike B, Reis-Filho JS. Metaplastic breast carcinomas are basal-like breast cancers: a genomic profiling analysis. Breast Cancer Res Treat. 2009;117(2):273–80. 18815879 10.1007/s10549-008-0197-9

[8] McMullen ER, Zoumberos NA, Kleer CG. Metaplastic breast carcinoma: update on histopathology and molecular alterations. Arch Pathol Lab Med. 2019;143(12):1492–6. 31765246 10.5858/arpa.2019-0396-RA

[9] Jung SY, Kim HY, Nam BH, Min SY, Lee SJ, Park C, et al. Worse prognosis of metaplastic breast cancer patients than other patients with triple-negative breast cancer. Breast Cancer Res Treat. 2010;120(3):627–37. 20143153 10.1007/s10549-010-0780-8

[10] Nelson RA, Guye ML, Luu T, Lai LL. Survival outcomes of metaplastic breast cancer patients: results from a US population-based analysis. Ann Surg Oncol. 2015;22(1):24–31. 25012264 10.1245/s10434-014-3890-4

[11] National Comprehensive Cancer Network (NCCN).Breast cancer (Version 4.2022). Available from: https://www.nccn.org/professionals/physician_gls/pdf/breast.pdf. Accessed 20 Oct 2022.

[12] Hennessy BT, Giordano S, Broglio K, Duan Z, Trent J, Buchholz TA, et al. Biphasic metaplastic sarcomatoid carcinoma of the breast. Ann Oncol. 2006;17:605–13. 16469754 10.1093/annonc/mdl006

[13] Chen IC, Lin CH, Huang CS, Lien HC, Hsu C, Kuo WH, et al. Lack of efficacy to systemic chemotherapy for treatment of metaplastic carcinoma of the breast in the modern era. Breast Cancer Res Treat. 2011;130(1):345–51. 21792625 10.1007/s10549-011-1686-9

[14] Aydiner A, Sen F, Tambas M, Ciftci R, Eralp Y, Saip P, et al. Metaplastic breast carcinoma versus triple negative breast cancer: survival and response to treatment. Medicine (Baltimore). 2015;94(52): e2341. 26717372 10.1097/MD.0000000000002341 PMC5291613

[15] Zhang Y, Lv F, Yang Y, Qian X, Lang R, Fan Y, et al. Clinicopathological features and prognosis of metaplastic breast carcinoma: experience of a major Chinese Cancer Center. PloS one. 2015;10(6): e0131409. 26115045 10.1371/journal.pone.0131409 PMC4482719

[16] Corso G, Frassoni S, Girardi A, De Camilli E, Montagna E, Intra M, et al. Metaplastic breast cancer: prognostic and therapeutic considerations. J Surg Oncol. 2021;123(1):61–70. 10.1002/jso.26248. 33047318 10.1002/jso.26248

[17] Cardoso F, Bedard PL, Winer EP, Pagani O, Senkus-Konefka E, Fallowfield LJ, et al. International guidelines for management of metastatic breast cancer: combination vs sequential single-agent chemotherapy. J Natl Cancer Inst. 2009;101:1174–81. 19657108 10.1093/jnci/djp235 PMC2736293

[18] Taube JH, Herschkowitz JI, Komurov K, Zhou AY, Gupta S, Yang J, et al. Core epithelial-to-mesenchymal transition interactome gene-expression signature is associated with claudin low and metaplastic breast cancer subtypes. Proc Natl Acad Sci U S A. 2010;107(35):15449–54. 20713713 10.1073/pnas.1004900107 PMC2932589

[19] Razavi P, Chang MT, Xu G, Bandlamudi C, Ross DS, Vasan N, et al. The genomic landscape of endocrine resistant advanced breast cancers. Cancer Cell. 2018;34(3):427-438.e426. 30205045 10.1016/j.ccell.2018.08.008 PMC6327853

[20] Ng CKY, Piscuoglio S, Geyer FC, Burke KA, Pareja F, Eberle CA, et al. The landscape of somatic genetic alterations in metaplastic breast carcinomas. Clin Cancer Res. 2017;23(14):3859–70. 28153863 10.1158/1078-0432.CCR-16-2857 PMC5511565

[21] Hennessy BT, Gonzalez-Angulo AM, Stemke-Hale K, Gilcrease MZ, Krishnamurthy S, Lee JS, et al. Characterization of a naturally occurring breast cancer subset enriched in epithelial-to-mesenchymal transition and stem cell characteristics. Cancer Res. 2009;69(10):4116–24. 19435916 10.1158/0008-5472.CAN-08-3441 PMC2737191

[22] Reis-Filho JS, Pinheiro C, Lambros MB, Milanezi F, Carvalho S, Savage K, et al. EGFR amplification and lack of activating mutations in metaplastic breast carcinomas. J Pathol. 2006;209(4):445–53. 16739104 10.1002/path.2004

[23] Dave B, Gonzalez DD, Liu ZB, Li X, Wong H, Granados S, Ezzedine NE, Sieglaff DH, Ensor JE, Miller KD, Radovich M. Role of RPL39 in metaplastic breast cancer. J Natl Cancer Inst. 2017;109(6):djw292. 10.1093/jnci/djw292 PMC6245334 28040796

[24] Afkhami M, Schmolze D, Yost SE, Frankel PH, Dagis A, Amanam IU, et al. Mutation and immune profiling of metaplastic breast cancer: correlation with survival. PLoS One. 2019;14(11): e0224726. 31693690 10.1371/journal.pone.0224726 PMC6834262

[25] Amin MB, Greene FL, Edge SB, Compton CC, Gershenwald JE, Brookland RK, Meyer L, Gress DM, Byrd DR, Winchester DP. The Eighth Edition AJCC Cancer Staging Manual: continuing to build a bridge from a population‐based to a more “personalized” approach to cancer staging. CA Cancer J Clin. 2017;67(2):93-9. 10.3322/caac.21388 28094848

[26] Goldhirsch A, Wood WC, Coates AS, Gelber RD, Thürlimann B, Senn HJ. Strategies for subtypes—dealing with the diversity of breast cancer: highlights of the St Gallen International Expert Consensus on the Primary Therapy of Early Breast Cancer 2011. Ann Oncol. 2011;22(8):1736–47. 21709140 10.1093/annonc/mdr304 PMC3144634

[27] Symmans WF, Peintinger F, Hatzis C, Rajan R, Kuerer H, Valero V, et al. Measurement of residual breast cancer burden to predict survival after neoadjuvant chemotherapy. J Clin Oncol. 2007;25(28):4414–22. 17785706 10.1200/JCO.2007.10.6823

[28] Cimino-Mathews A, Verma S, Figueroa-Magalhaes MC, Jeter SC, Zhang Z, Argani P, et al. A clinicopathologic analysis of 45 patients with metaplastic breast carcinoma. Am J Clin Pathol. 2016;145(3):365–72. 27124919 10.1093/ajcp/aqv097

[29] El Zein D, Hughes M, Kumar S, Peng X, Oyasiji T, Jabbour H, et al. Metaplastic carcinoma of the breast is more aggressive than triple-negative breast cancer: a study from a single institution and review of literature. Clin Breast Cancer. 2017;17(5):382–91. 28529029 10.1016/j.clbc.2017.04.009 PMC5537027

[30] Tadros AB, Sevilimedu V, Giri DD, Zabor EC, Morrow M, Plitas G. Survival outcomes for metaplastic breast cancer differ by histologic subtype. Ann Surg Oncol. 2021;28(8):4245–53. 33389291 10.1245/s10434-020-09430-5

[31] Song Y, Liu X, Zhang G, Song H, Ren Y, He X, et al. Unique clinicopathological features of metaplastic breast carcinoma compared with invasive ductal carcinoma and poor prognostic indicators. World J Surg Oncol. 2013;11(1):1–9. 23738706 10.1186/1477-7819-11-129 PMC3679991

[32] Leyrer CM, Berriochoa CA, Agrawal S, Donaldson A, Calhoun BC, Shah C, et al. Predictive factors on outcomes in metaplastic breast cancer. Breast Cancer Res Treat. 2017;165(3):499–504. 28689362 10.1007/s10549-017-4367-5

[33] Takala S, Heikkilä P, Nevanlinna H, Blomqvist C, Mattson J. Metaplastic carcinoma of the breast: prognosis and response to systemic treatment in metastatic disease. Breast J. 2019;25(3):418–24. 30925636 10.1111/tbj.13234

[34] Polamraju P, Haque W, Cao K, Verma V, Schwartz M, Klimberg VS, et al. Comparison of outcomes between metaplastic and triple-negative breast cancer patients. Breast. 2020;49:8–16. 31675684 10.1016/j.breast.2019.10.003 PMC7375639

[35] Zhang J, Yang C, Lei C, Zhang Y, Ji F, Gao H, et al. Survival outcomes after breast-conserving therapy compared with mastectomy for patients with early-stage metaplastic breast cancer: a population-based study of 2412 patients. Breast. 2021;58:10–7. 33878598 10.1016/j.breast.2021.03.010 PMC8080072

[36] Xia L-Y, Xu W-Y, Hu Q-L. The different outcomes between breast-conserving surgery plus radiotherapy and mastectomy in metaplastic breast cancer: a population-based study. PLoS ONE. 2021;16(9): e0256893. 34473783 10.1371/journal.pone.0256893 PMC8412345

[37] Haque W, Verma V, Schwartz MR, Lim B, Mangalampalli N, Butler EB, Teh BS. Neoadjuvant chemotherapy for metaplastic breast cancer: response rates, management, and outcomes. Clin Breast Cancer. 2022;22(5):e691–9. 10.1016/j.clbc.2022.01.006. (Epub 2022 Jan 31 PMID: 35193807). 35193807 10.1016/j.clbc.2022.01.006

[38] Rakha EA, Tan PH, Varga Z, Tse GM, Shaaban AM, Climent F, et al. Prognostic factors in metaplastic carcinoma of the breast: a multi-institutional study. Br J Cancer. 2015;112(2):283–9. 25422911 10.1038/bjc.2014.592 PMC4453452

[39] Erjan A, Almasri H, Abdel-Razeq H, Al-Masri M, Haddad H, Alnsour A, et al. Metaplastic breast carcinoma: experience of a Tertiary Cancer Center in the Middle East. Cancer Control. 2021;28:10732748211004888. 10.1177/10732748211004889. 33827281 10.1177/10732748211004889 PMC8204524

[40] Ong CT, Campbell BM, Thomas SM, Greenup RA, Plichta JK, Rosenberger LH, et al. Metaplastic breast cancer treatment and outcomes in 2500 patients: a retrospective analysis of a National Oncology Database. Ann Surg Oncol. 2018;25(8):2249–60. 29855830 10.1245/s10434-018-6533-3 PMC6039971

[41] Basho RK, Gilcrease M, Murthy RK, Helgason T, Karp DD, Meric-Bernstam F, et al. Targeting the PI3K/AKT/mTOR pathway for the treatment of mesenchymal triple-negative breast cancer: evidence from a phase 1 trial of mTOR inhibition in combination with liposomal doxorubicin and bevacizumab. JAMA Oncol. 2017;3(4):509–15. 27893038 10.1001/jamaoncol.2016.5281

[42] Kwa MJ, Iwano A, Esteva FJ, Novik Y, Speyer JL, Oratz R,et al. Phase II trial of pembrolizumab in combination with nab-paclitaxel in patients with metastatic HER2-negative breast cancer.JCO.2017.35.15_suppl TPS1124, 10.1200/JCO.2017.35.

[43] Sylvia Adams. Dramatic response of metaplastic breast cancer to chemo-immunotherapy. NPJ Breast Cancer. 2017;3:8. 10.1038/s41523-017-0011-0.10.1038/s41523-017-0011-0 PMC5445614 28649648

[44] Adams S, Othus M, Patel SP, Miller KD, Chugh R, Schuetze SM, et al. A multicenter phase II trial of ipilimumab and nivolumab in unresectable or metastatic metaplastic breast cancer: cohort 36 of dual anti–CTLA-4 and anti–PD-1 blockade in rare tumors (DART, SWOG S1609) Ipilimumab and nivolumab in rare tumors S1609: metaplastic. Clin Cancer Res. 2022;28(2):271–8. 10.1158/1078-0432.CCR-21-2182. 34716198 10.1158/1078-0432.CCR-21-2182 PMC8776596

